# Intrapericardial Pulmonary Vein Ligation to Prevent Stump Thrombosis During Left Upper Lobectomy

**DOI:** 10.1016/j.atssr.2024.04.032

**Published:** 2024-05-27

**Authors:** Shunta Ishihara, Masanori Shimomura, Hiroaki Tsunezuka, Satoru Okada, Tatsuo Furuya, Tatsuya Yoshikawa, Masayoshi Inoue

**Affiliations:** 1 Division of Thoracic Surgery, Department of Surgery, Graduate School of Medical Science, Kyoto Prefectural University of Medicine, Kyoto, Japan; 2 Department of Thoracic Surgery, Otsu City Hospital, Shiga, Japan; 3 Department of Radiology, Graduate School of Medical Science, Kyoto Prefectural University of Medicine, Kyoto, Japan

## Abstract

**Background:**

Postoperative cerebral infarction, a serious complication of surgery, is occasionally experienced with pulmonary vein stump thrombosis (PVST), which is frequently observed after left upper lobectomy (LUL). Herein, we prospectively investigated whether PVST could be safely prevented by intrapericardial ligation of the superior pulmonary vein (SPV) to shorten the SPV stump during LUL.

**Methods:**

In a consecutive 21 patients who underwent LUL, we ligated the proximal intrapericardial SPV with 1-0 silk suture and divided the distal hilar SPV by an automatic stapling device. We measured the SPV stump length from the left atrium to the point of ligation and evaluated the presence of PVST on postoperative computed tomography. The procedure time was measured as the time from pericardial treatment initiation to the distal SPV division. Furthermore, the safety of the procedure and postoperative complications were evaluated and compared with those of 76 historical control patients who underwent LUL without intrapericardial SPV ligation.

**Results:**

The median procedure time was 8.8 minutes, and the median blood loss was 3 g. The median length of the SPV stump after the procedure was 5.0 mm. The 30- and 90-day mortality rates were both 0% for patients who underwent LUL with SPV ligation. None of the patients in the SPV ligation group showed signs of PVST on postoperative contrast-enhanced computed tomography images or had cerebrovascular disease. No significant difference in postoperative complications was observed between the groups.

**Conclusions:**

Intrapericardial SPV ligation is safe and has a potential to prevent cerebral infarction after LUL.

## Visual Abstract

**Figure:**
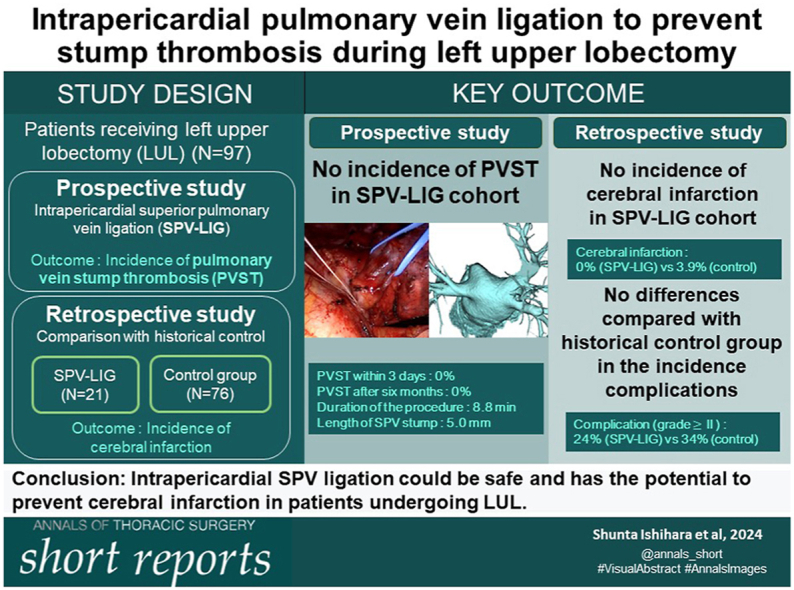

**In Short**

▪ The intrapericardial superior pulmonary vein (SPV) ligation procedure to prevent pulmonary vein stump thrombosis during left upper lobectomy took 8.8 minutes and shortened the SPV stump to 5.0 mm.
▪ None of the patients with intrapericardial SPV ligation showed pulmonary vein stump thrombosis or had cerebrovascular disease.
▪ Intrapericardial SPV ligation could be safe and has the potential to prevent cerebral infarction in patients undergoing left upper lobectomy.

The incidence of postoperative cerebral infarction is higher after left upper lobectomy (LUL) than after other lobectomies and ranges from 1.5% to 4.2%.^1^^,^^2^ The intrapericardial stump of the left upper pulmonary vein is anatomically longer than that of other pulmonary veins, resulting in thrombus formation in the stump. LUL is therefore more likely to cause pulmonary vein stump thrombosis (PVST) than other types of lobectomies.^3^ The incidence of PVST after LUL is reported to be approximately 7.0% to 30.8%^3^^,^^4^ and persists in the late phase after surgery (>30 days); PVST is a suspected cause of post-LUL cerebral infarction.^1^ Previous studies, including a large retrospective study,^1^^,^^5^ a real-world database study,^6^ and a multicenter study,^2^ demonstrated a higher incidence of cerebral infarction with LUL. Countermeasures against PVST or cerebral infarction during LUL are therefore important. We hypothesized that shortening the intrapericardial pulmonary vein stump would decrease PVST and thus prevent stroke onset. Thus, in this study, we investigated whether intrapericardial superior pulmonary vein (SPV) ligation could prevent PVST after LUL and also assessed the safety.

## patients and Methods

### Study Design

Patients who underwent LUL and left pneumonectomy at the University Hospital, Kyoto Prefectural University of Medicine between January 2015 and September 2022 were enrolled in this study, which was approved by the institutional review board of Kyoto Prefectural University of Medicine (ERB-C-2438).

With a view to preventing cerebral infarction, we performed intrapericardial ligation of the SPV during LUL in patients operated on between December 2020 and September 2022. This preliminary study was performed to prospectively evaluate the safety of intrapericardial ligation of SPV. The primary outcome was the presence of PVST detected by contrast-enhanced computed tomography (CT) scanning. The study protocol is shown in the Supplemental Material. The surgical technique of SPV ligation is shown in the Video, and a representative thoracoscopic procedure is shown in Figure A. The pericardium was incised at the root end of the SPV, and the proximal side of the SPV was ligated within the pericardial sac with a 1-0 silk suture. The distal SPV was divided with an automatic stapling device.

**Figure:**
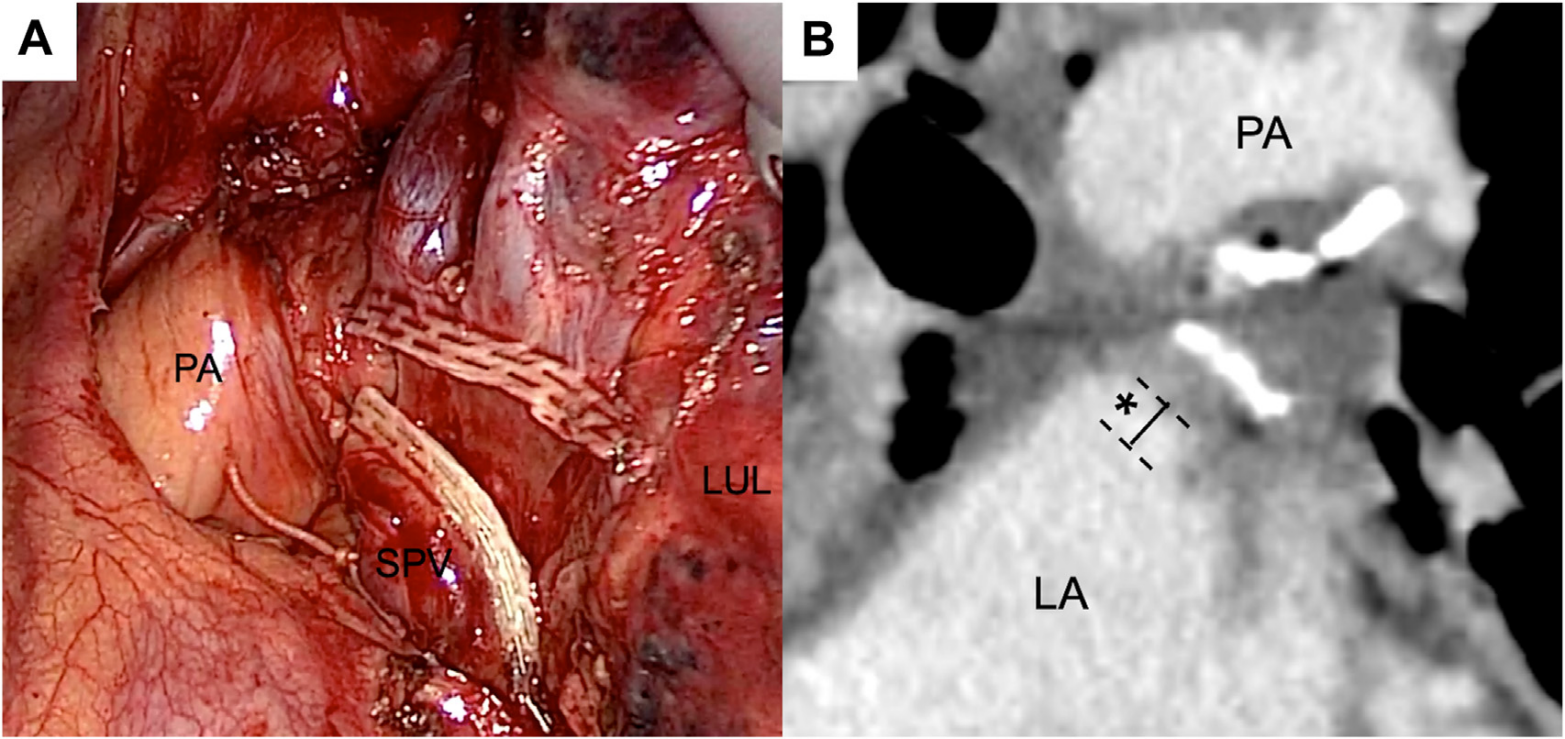
(A) Postprocedure image of superior pulmonary vein (SPV) ligation. (B) The length of the SPV stump was defined as the distance between the left atrium (LA) and the point of ligation (∗). (LUL, left upper lobe; PA, pulmonary artery.)

Evaluation of the SPV ligation procedure was performed by the radiologist (T.Y.) who was blinded to the clinical information. This evaluation comprised measurement of the length of the SPV stump between the SPV orifice into the left atrium and the point of ligation in 3 spatial planes (axial, coronal, and sagittal) of the postoperative contrast-enhanced CT image (Figure B). The secondary outcome was the occurrence of postoperative cerebral infarction. The surgical procedure was evaluated by assessing the occurrence of intraoperative complications, duration of the procedure, and length of the SPV stump. Patients’ clinical information (clinical background, surgical information, and postoperative complications including cerebral infarction) and perioperative serum D-dimer levels were recorded.

To evaluate the safety of the procedure, we retrospectively compared the complications in the SPV ligation group with those of a historical conventional stapling (control) group composed of patients who underwent LUL between January 2015 and December 2020. We investigated the rates of postoperative complications associated with the SPV ligation and non-SPV treatment (historical control) groups.

### Statistical analyses

Categorical variables were analyzed by Pearson *χ*^2^ test. Continuous variables were analyzed by the Wilcoxon signed rank test. Statistical significance was set at *P* < .05. The results for each continuous variable are presented as the median and range or interquartile range. Statistical analyses were performed with JMP software (version 12.2; SAS Institute).

## Results

### Surgical Procedure and Outcome of SPV Ligation

Twenty-one patients who underwent LUL with intrapericardial SPV ligation (SPV ligation group) were enrolled; 1 patient who underwent LUL with intrapericardial SPV stapling without SPV ligation was excluded. The clinical characteristics of the 21 enrolled patients are presented in Supplemental Table 1.

The SPV ligation procedure and postoperative outcomes are shown in Table 1. The median procedure time of the SPV ligation was 8.8 minutes (range, 4.6-27.0 minutes), and the median length of the SPV stump was 5.0 mm (range, 0-26.4 mm). Focusing on the surgical approach, the median lengths of the SPV stump in robot-assisted thoracoscopic surgery (RATS) were longer than those in thoracoscopic and open thoracotomy. Postoperative contrast-enhanced CT images exhibited no thrombus in the left atrium in the early or the late phase in any of the patients. No cerebral infarction occurred. Both the 30- and 90-day mortality rates were 0%.

**Table 1:** Clinical Data of SPV Ligation Procedure and Postoperative Outcomes

Variable	Median (range) or No. (%)
Duration of SPV ligation procedure, min	8.8 (4.6-27.0)
Surgical incision, mm	5.0 (3.0-20.0)
Length of SPV stump, mm	
All	5.0 (0-26.4)
Thoracoscopic approach	2.5 (0-25.0)
Open thoracotomy approach	3.3 (0-14.9)
Robot-assisted approach	17.1 (0-26.4)
Preoperative serum D-dimer level, μg/mL	0.6 (IQR, 0.5-1.1)
Postoperative serum D-dimer level, μg/mL	2.3 (IQR, 1.3-5.3)
Postoperative infarction	0 (0)
Intra-atrium thrombus in early phase (POD 1, 8 patients; POD 2, 8 patients; POD 3, 5 patients)	0 (0)
Intra-atrium thrombus in late phase	0 (0)

IQR, interquartile range; POD, postoperative day; SPV, superior pulmonary vein.

### Comparison With Historical Control

Seventy-six patients underwent LUL or left pneumonectomy without SPV ligation (historical control group). The clinical characteristics and postoperative complications of the SPV ligation and control groups are displayed in Table 2. In the SPV ligation group, postoperative complications occurred in 5 patients (24%): 5 patients (24%) with atrial fibrillation and 1 patient (5%) with pneumonia. None of the SPV ligation patients had cerebral infarction (0%), whereas 3 patients had cerebral infarction in the control group (3.9%). The characteristics of the patients with cerebral infarction are shown in Supplemental Table 2. One patient in the control group (case 3), in whom cerebral infarction had occurred on postoperative day 2, had PVST 2 months later (Supplemental Figure). Compared with the control group, the SPV ligation group showed no significant differences in the incidence of complications, atrial fibrillation, or cerebral infarction. The median hospital stay was significantly longer in the SPV ligation group than in the control group; however, there was no difference in the median duration of chest drainage and the amount of chest drainage (postoperative day 1) between the SPV ligation and control groups.

**Table 2:** Characteristics and Postoperative Complications of SPV Ligation Compared With Control Group

Characteristics	SPV Ligation (N = 21)	Control (N = 76)	*P* values
Age, y	74.0 (63-80)	68.0 (64-74)	.10^a^
Sex, male	13 (62)	49 (64)	.83^a^
Surgical approach			.02^b^
Open thoracotomy	5 (24)	19 (25)	
Thoracoscopic	11 (52)	55 (72)	
Robot assisted	5 (24)	2 (3)	
Operative time, min	227 (196-261)	246 (196-286)	.39^a^
Bleeding, g	3 (3-71)	12 (3-100)	.26^a^
Pathologic size, cm	2.5 (1.9-4.1)	2.1 (1.3-3.5)	.18^a^
Pathologic stage of lung cancer (≥IB)	10 (53)	34 (52)	.93^b^
Preoperative serum D-dimer level, μg/mL	0.6 (0.5-1.1)	0.5 (0.5-0.9)	.76^a^
Preoperative serum CRP level, mg/mL	0.09 (0.04-0.27)	0.08 (0.04-0.38)	.79^a^
Complication (≥grade II)			
All	5 (24)	26 (34)	.34^b^
Atrial fibrillation	5 (24)	10 (14)	.26^b^
Cerebral infarction	0 (0)	3 (3.9)	.36^b^
Other	1 (5)	14 (18)	.13^b^
Postoperative serum CRP level, mg/mL			
POD 4	7.46 (6.08-13.75)	6.82 (4.81-10.97)	.24^a^
≥POD 7	3.12 (0.41-5.61)	2.78 (1.51-4.65)	.91^a^
Duration of hospital stay, d	10 (9-14)	9 (7-11)	.01^a^
Amount of chest drainage (POD 1), mL/d	350 (225-400)	255 (173-355)	.13^a^
Duration of chest drainage, d	3 (2-4)	3 (2-4)	.12^a^

Data are presented as median (interquartile range) or number (%).

CRP, C-reactive protein; POD, postoperative day; SPV, superior pulmonary vein.

a Pearson *χ*^2^ test.

b Wilcoxon rank sum test.

## Comment

This study confirms the safety and efficacy of intrapericardial SPV ligation for prevention of PVST causing postoperative cerebral infarction. Twenty-one patients underwent LUL with intrapericardial SPV ligation, and no intraoperative serious bleeding or other procedural complications occurred. The SPV ligation procedure required 8.8 minutes, suggesting that it is practically feasible. Furthermore, neither PVST nor cerebral infarction incident after LUL with intrapericardial SPV ligation suggests the clinical efficacy for prevention of postoperative cerebral infarction.

Thrombus development in the resected pulmonary vein stump may be involved,^1^ as the intrapericardial length of the left SPV is anatomically longer than that of the other pulmonary veins. Dynamic blood movement analysis revealed that PVST was accelerated after LUL.^7^ Ohtaka and colleagues^3^ reported PVST in 3.6% of patients after lobectomy, with a higher incidence of 13.5% in the left upper lobe. Hattori and colleagues,^4^ in their prospective observational study, reported the presence of a thrombus in the SPV stump in 30.8% of patients who underwent LUL, and thrombus formation occurred within 1 week postoperatively in most cases. Nakano and colleagues^8^ reported that the PVST after LUL was released from the pulmonary vein stump and caused cerebral infarction. In our historical group, 3 cases had postoperative cerebral infarction in the early phase (Supplemental Table 2). Based on the enhanced CT, these patients did not have PVST, and cerebral infarction may have developed after release of the PVST. The onset of stroke after LUL could occur in the early postoperative period^2^; therefore, the thrombus should be detected early postoperatively for preventive management of cerebral complication. In this study, the absence of a thrombus in the atrium and cerebral infarction after intrapericardial SPV ligation was confirmed by contrast-enhanced CT within 3 days and 6 months after surgery. Thus, we deduce that intrapericardial SPV ligation has the potential to prevent PVST after LUL.

The SPV stump length was shortened to 5.0 mm by intrapericardial SPV ligation and further shortened to 2.5 mm after thoracoscopic surgery; this reduction could contribute to the prevention of PVST. Ohtaka and colleagues^3^ reported that the median length of the left SPV stump was 17 mm, whereas that of other pulmonary vein stumps was approximately 5 mm; hence, the intrapericardial SPV ligation is sufficient to reduce the risk to a level similar to that for resection of other lung lobes. It has been proposed that the left SPV should be divided during LUL to a length as short as possible for the prevention of thrombus formation.^9^ Our data showed that the RATS approach left a longer SPV stump. RATS may complicate feeding of the ligature into the intrapericardial sac or not apply sufficient force to the larger vessels. RATS without tactile sensation requires careful manipulation for intrapericardial SPV ligation.

Compared with the conventional treatment, our method of intrapericardial SPV ligation revealed no increase in the occurrence of severe complications. Proximal SPV ligation, including that of the pericardial sac, reportedly causes bleeding or cardiac tamponade,^10^ which was not observed in this cohort. The rate of atrial fibrillation and the amount of chest drainage (postoperative day 1) slightly increased (not significant), and the length of hospital stay was prolonged in the SPV ligation group compared with the control group. These may be possible adverse effects related to intrapericardial SPV ligation.

The primary limitation of this study is that we did not consider cerebral infarction after lobectomies other than LUL, such as left lower lobectomy and segmentectomy,^4^ although it has a low incidence. Another limitation is the small sample size, which may be insufficient to show whether this method prevents cerebral infarction after LUL.

In conclusion, intrapericardial SPV ligation could be safe and has the potential to prevent cerebral infarction in patients undergoing LUL. This procedure may be a feasible option that involves minimally invasive surgery because no other prophylaxis is currently available.
