# Supplementary material for: Intrapericardial Pulmonary Vein Ligation to Prevent Stump Thrombosis During Left Upper Lobectomy
Source: Ann Thorac Surg Short Rep. 2024 May 27;2(4):608–12. doi: 10.1016/j.atssr.2024.04.032 (PMC11708331; doi:10.1016/j.atssr.2024.04.032)
Supplement: Supplementary Tables 1 and 2 [file mmc1.pptx]

## Slide 1
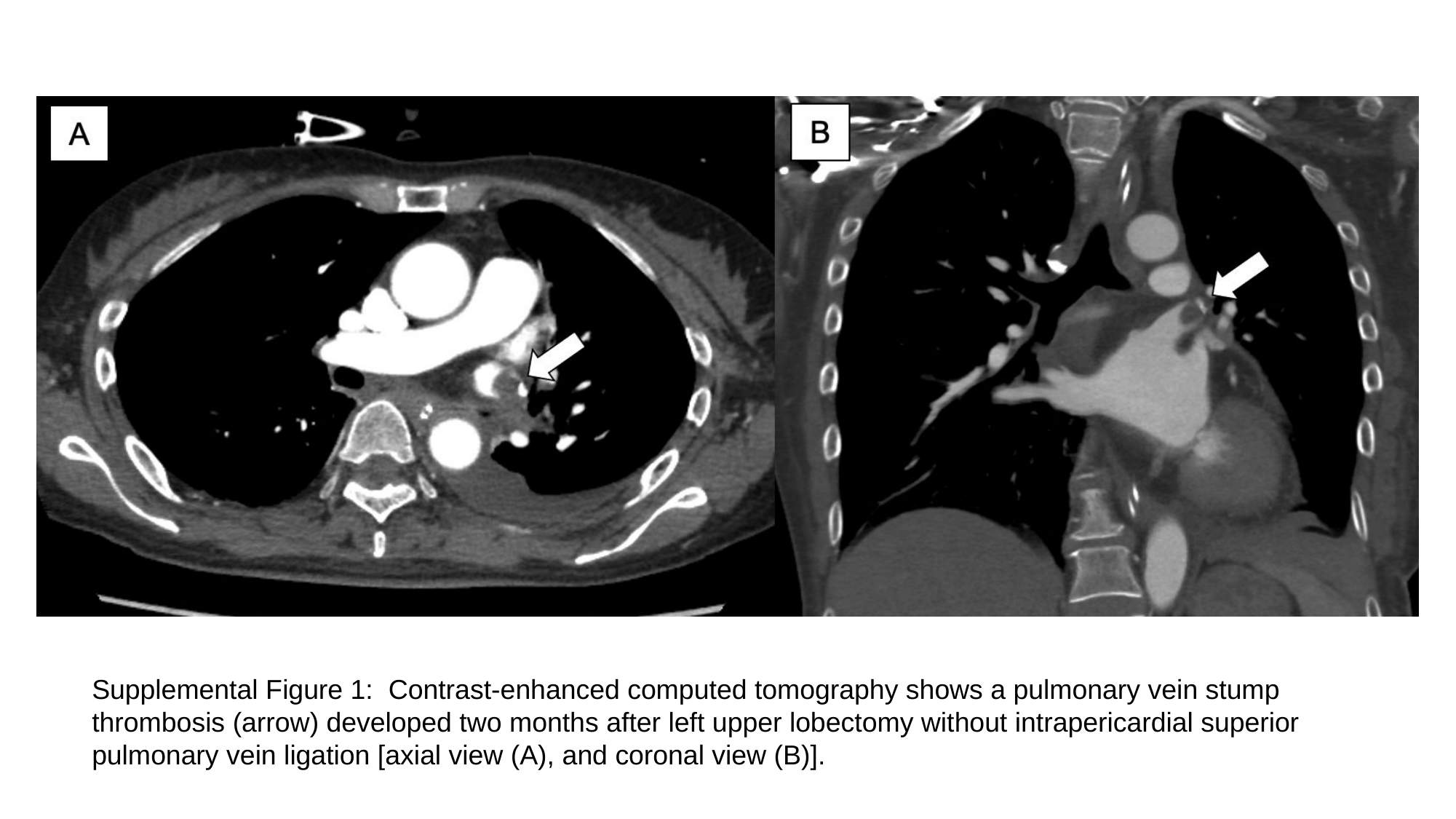

Supplemental Figure 1: Contrast-enhanced computed tomography shows a pulmonary vein stump thrombosis (arrow) developed two months after left upper lobectomy without intrapericardial superior pulmonary vein ligation [axial view (A), and coronal view (B)].
