# Supplementary material for: Intrapericardial Pulmonary Vein Ligation to Prevent Stump Thrombosis During Left Upper Lobectomy
Source: Ann Thorac Surg Short Rep. 2024 May 27;2(4):608–12. doi: 10.1016/j.atssr.2024.04.032 (PMC11708331; doi:10.1016/j.atssr.2024.04.032)
Supplement: Supplementary Figure [file mmc2.docx]

**Supplemental Table 1. Characteristics of 21 patients with SPV ligation**

| Characteristics | Median (IQR) or N (%) |
| --- | --- |
| Age (years) | 74.0 (63–80) |
| Sex (male) | 13 (62) |
| Diagnosis |  |
| Primary lung cancer | 19 (90) |
| Metastatic lung tumor | 2 (10) |
| Pathological stage of lung cancer (≥IB) | 10 (53) |
| Preoperative comorbidities |  |
| Atrial fibrillation | 3 (14) |
| Diabetes mellitus | 2 (10) |
| Cerebral infarction | 2 (10) |
| Surgical approach |  |
| Open thoracotomy | 5 (24) |
| Thoracoscopic | 11 (52) |
| Robot-assisted | 5 (24) |
| Mode of surgery |  |
| Lobectomy | 18 (86) |
| Pneumonectomy | 2 (10) |
| Sleeve-lobectomy | 1 (5) |

*IQR,* interquartile range; *SPV,* superior pulmonary vein

**Supplemental Table 2. Characteristics of patients with postoperative cerebral infarction after left upper lobectomy**

| Case | Age (years) | Sex | Approach | Operative  time (min) | Blood loss (g) | Tumor size (cm) | P-stage of lung cancer | Interval (days) | Concomitant conditions | Presence of the PVST | UFH dose |
| --- | --- | --- | --- | --- | --- | --- | --- | --- | --- | --- | --- |
| 1 | 58 | Male | TS | 187 | 3 | 4.5 | IIA | 1 | None | NA | 10,000 IU/day |
| 2 | 74 | Male | TS | 181 | 5 | 2.8 | IB | 0 | Hypertension | None | none |
| 3 | 58 | Female | TS | 209 | 3 | 1.3 | IA2 | 2 | None | None | 10,000 IU/day |

*NA,* not assessed; *PVST,* pulmonary vein stump thrombosis; *TS*, thoracoscopic surgery; *UFH*, unfractionated heparin.
